# Systemic administration of the PRMT5 inhibitor GSK3326595 does not induce overt cardiotoxicity in the murine heart

**DOI:** 10.1016/j.jmccpl.2026.100863

**Published:** 2026-08-15

**Authors:** Victoria Mauz, Clara Steinacher, Jürgen Burhenne, Kevin Steimel, Christina Mertens, Martina U. Muckenthaler, Matthias Dewenter, Johannes Backs

**Affiliations:** a Heidelberg University, Medical Faculty Heidelberg, Institute of Experimental Cardiology, 69120, Heidelberg, Germany; b Heidelberg University Hospital, Department of Internal Medicine VIII, 69120, Heidelberg, Germany; c German Center for Cardiovascular Research (DZHK), Partner Site Heidelberg/Mannheim, 69120, Heidelberg, Germany; d Molecular Medicine Partnership Unit, European Molecular Biology Laboratory (EMBL), 69117, Heidelberg, Germany and Heidelberg University, 69120, Heidelberg, Germany; e Heidelberg University, Medical Faculty Heidelberg, Heidelberg University Hospital, Internal Medicine IX - Department of Clinical Pharmacology and Pharmacoepidemiology, 69120, Heidelberg, Germany; f Center for Translational Biomedical Iron Research, Department of Pediatric Oncology, Immunology, and Hematology, University of Heidelberg, Heidelberg, Germany; g Helmholtz-Institute for Translational AngioCardioScience (HI-TAC) of the Max Delbrück Center for Molecular Medicine in the Helmholtz Association (MDC) at Heidelberg University, Heidelberg, 69117, Germany

**Keywords:** PRMT5, Cardiooncology, Cardiotoxicity

## Abstract

Protein Arginine Methyltransferase (PRMT5) has emerged as a promising target for antineoplastic strategies. Given its oncogenic properties in promoting tumorigenesis and metastasis of several cancer entities, pharmacological approaches to inhibit the enzymatic activity of PRMT5 (PRMT5i) have gained particular attention. Among others, GSK3326595 is currently evaluated in clinical studies for the treatment of hematological malignancies. However, besides the pathophysiological relevance, PRMT5 is a ubiquitously expressed enzyme with essential cellular functions. Previous studies uncovered a substantial role in cardiomyocytes along with heart failure caused by loss of PRMT5 raising the question if PRMT5i may exert adverse effects on the cardiovascular system. Thus, we conducted comprehensive pharmacokinetic profiling of GSK3326595 in mice. We found that systemic administration leads to a homogenous distribution across organs and tissues. While major depression of hematopoiesis could be detected, we did not find detrimental impact on systolic function, cardiac morphology and homeostasis indicating differential pharmacodynamics on proliferating and postmitotic cells. In conclusion, systemic treatment with GSK3326595 shows no overt effects on the murine heart under the conditions tested in this study.

## Highlights

• PRMT5 inhibition by GSK3326595 does not induce overt cardiotoxicity in mice.
• GSK3326595 is systemically distributed with major targeting of liver and kidney.
• GSK3326595 strongly depresses hematopoiesis in mice.

## Graphical abstract

**Figure:**
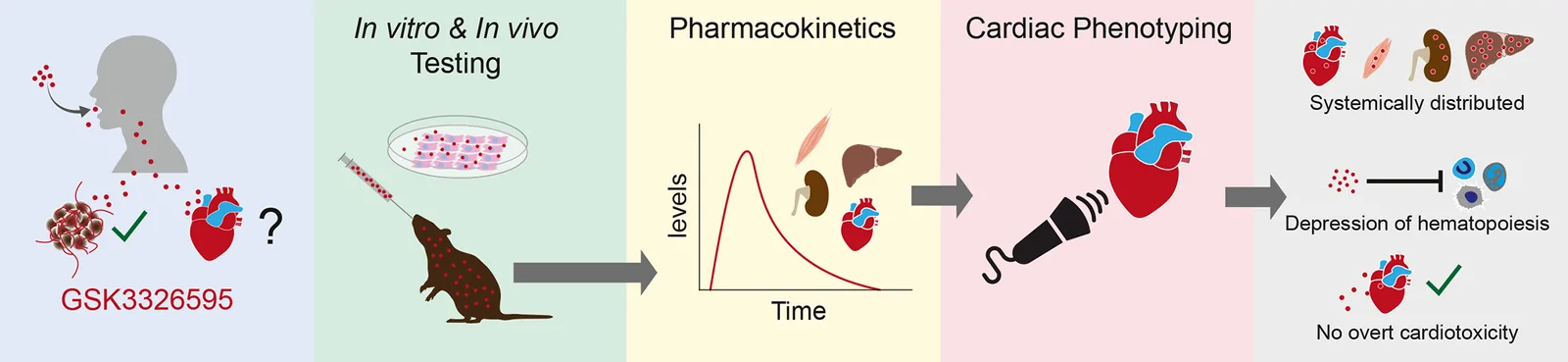

## 1 Introduction

Methylation of arginine represents a posttranslational modification regulating diverse cellular processes including differentiation and proliferation [1]. Involved enzymes are Protein Arginine Methyltransferases (PRMTs) subclassified in 3 entities depending on catalytic properties. PRMT5 is the main representative of type II enzymes promoting monomethylation and symmetric dimethylation of arginine residues (SDMA) [2]. Along with substrates such as EGFR [3] and KLF4 [4], PRMT5 is well characterized as an oncogene whose expression and activity correlates with mortality and poor prognosis of several tumor entities [5], [6]. Accordingly, PRMT5 has gained attention as novel target for antineoplastic strategies and pharmacological inhibition (PRMT5i) has proven to sufficiently attenuate growth and improve survival in experimental models of Mantle cell lymphoma, pancreatic and liver cancer [7], [8], [9]. Based on EPZ015666 as a potent substrate-competitive inhibitor, several molecules have been developed targeting substrate or cofactor binding [10] and entered clinical studies [11]. Among them, GSK3326595 is tested against myeloid neoplasms [12], breast cancer (Clinicaltrials.gov; NCT04676516), solid cancer and Non-Hodgkin's Lymphoma (NCT02783300).

In contrast to malignancies, the physiological role of PRMT5 in adult organisms is less well understood but includes essential functions during cell differentiation and tissue homeostasis [6]. Recently, PRMT5 was attributed a critical role in cardiomyocytes as a regulator of O-GlcNAcylation [13]. Using genetic models of conditional *Prmt5* deletion in mice (*Prmt5*-cKO), loss of PRMT5 in cardiomyocytes induced progressive systolic dysfunction, pro-fibrotic remodeling and increased mortality. Hence, PRMT5 activity is not only sufficient to induce malignant transformation, progression and metastasis, it is also required for maintaining cardiac integrity indicating differential downstream signaling in mitotic and predominantly post-mitotic cells. Targeting methyltransferase activity therefore needs to be considered comprehensively and potential adverse effects, particularly on the heart are to be evaluated.

Here, we report that GSK3326595 is distributed across all organs tested including liver, kidney, skeletal muscle and heart upon oral and intravenous application in mice. We found a rapid dose-dependent maximum 1 h post administration along with an exponential clearance within 12 h. Whereas liver and kidney showed the highest peak concentration, the heart was targeted by 26.0% and 19.0% dosage on average, respectively. Despite its pharmacokinetic targeting, long-term administration did not induce overt effects on cardiac homeostasis as determined by functional, histological and molecular parameters. In contrast, hematopoiesis was strongly affected leading to anemia and leucopenia suggesting major impact on highly proliferative systems.

## 2 Results

### 2.1 PRMT5 inhibition *in vitro*

**Fig. 1:**
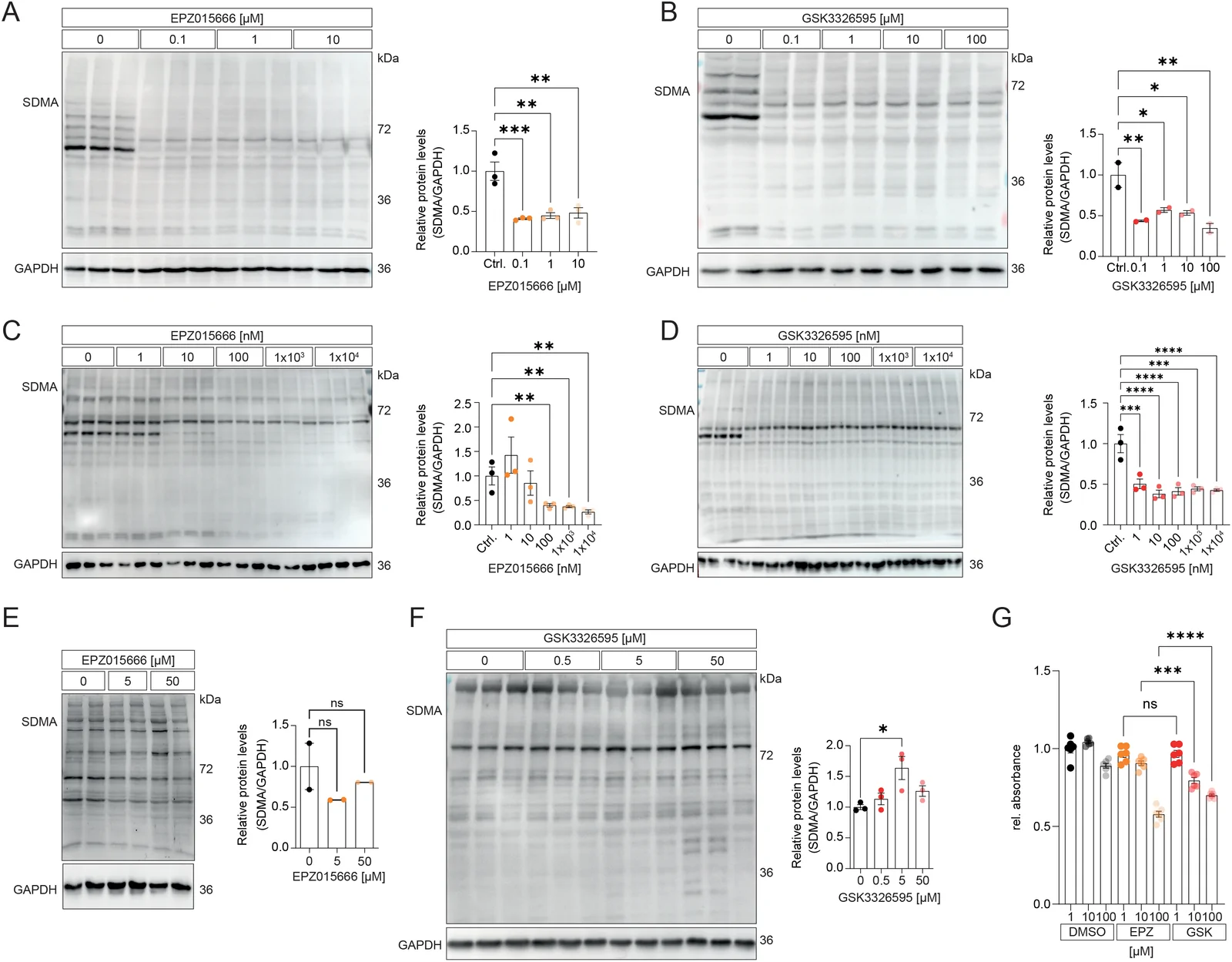
PRMT5 inhibition *in vitro*. (A-D) Immunoblot analyses of HEK293 cells treated with EPZ015666 or GSK3326595 at indicated dosages for 96 h. Global levels of symmetrically dimethylated arginine (SDMA) were used as readout for enzymatic activity. GAPDH served as loading control. *n* = 3 replicates per group (A, C, D), *n* = 2 replicates per group (B). Each experiment was performed at least 3 times (independent cell passages) along with at least 2 technical replicates (individual wells) per dosage. Statistical analysis: values are presented as Mean ± SEM (A) ***p = 0.0008, **p = 0.0012 (1 μM), **p = 0.0018 (10 μM), (B) **p = 0.0091 (0.1 μM), *p = 0.0275 (1 μM), *p = 0.0203 (10 μM), **p = 0.0048 (100 μM), (C) **p = 0.0057 (100 nM), **p = 0.0045 (1000 nM), **p = 0.0017 (10,000 nM) (D) ***p = 0.0003 (1 nM), ***p = 0.0001 (1000 nM), ****p < 0.0001 (Ordinary one-way ANOVA and Dunnett‘s multiple comparisons test). (E, F) Immunoblot analyses of neonatal rat ventricular cardiomyocytes treated with EPZ015666 or GSK3326595 at indicated dosages. n = 2 replicates per group (E), *n* = 3 replicates per group (F). Statistical analysis: values are presented as Mean ± SEM *p = 0.0113 (Ordinary one-way ANOVA and Dunnett‘s multiple comparisons test). Each experiment was performed at least 3 times (independent cell preparations) along with at least 2 technical replicates (individual wells) per dosage. (G) Cytotoxicity assessment upon PRMT5i exposure for 48 h. Each value represents a separately treated well. The experiment was performed 3 times using different cell passages along with 6 technical replicates (individual wells) per dosage. Statistical analysis: values are presented as Mean ± SEM ***p = 0.0003, ****p < 0.0001 (Ordinary one-way ANOVA and Bonferroni‘s multiple comparisons test).

In order to characterize pharmacodynamic properties of GSK3326595, we first evaluated its potential to inhibit PRMT5 in comparison to the well characterized molecule EPZ015666 *in vitro*. We used the posttranslational modification SDMA as global readout of PRMT5 activity [7]. Both inhibitors showed strong overall reduction of SDMA in HEK293 cells indicating efficient uptake and strong pharmacodynamic effects in the micromolar range (Fig. 1A, B). To further compare dose-dependent effects we extended our approach to minimal dosages as of 1 nM. Here, GSK3326595 was already sufficient to reduce global SDMA in contrast to EPZ015666 (Fig. 1C, D). In agreement with published data, GSK3326595 was overall more effective as compared to IC_50_ in biochemical assays (22 nM for EPZ015666 [7]; 6.2 nM for GSK3326595 [14]). In parallel, both compounds were examined in neonatal rat cardiomyocytes. Of note, using concentrations up to 50 μM we did not detect a significant reduction of global SDMA (Fig. 1E, F) implying a different pharmacokinetic and/or pharmacodynamic profile in primary cardiomyocytes compared to an immortalized cell line. Despite higher effectivity of GSK3326595 in HEK293 cells, dosage escalation beyond 10 μM revealed significantly less cytotoxicity as compared to EPZ015666 (Fig. 1G) providing the rationale for further *in vivo* administration.

### 2.2 Pharmacokinetics of GSK3326595 *in vivo*

**Fig. 2:**
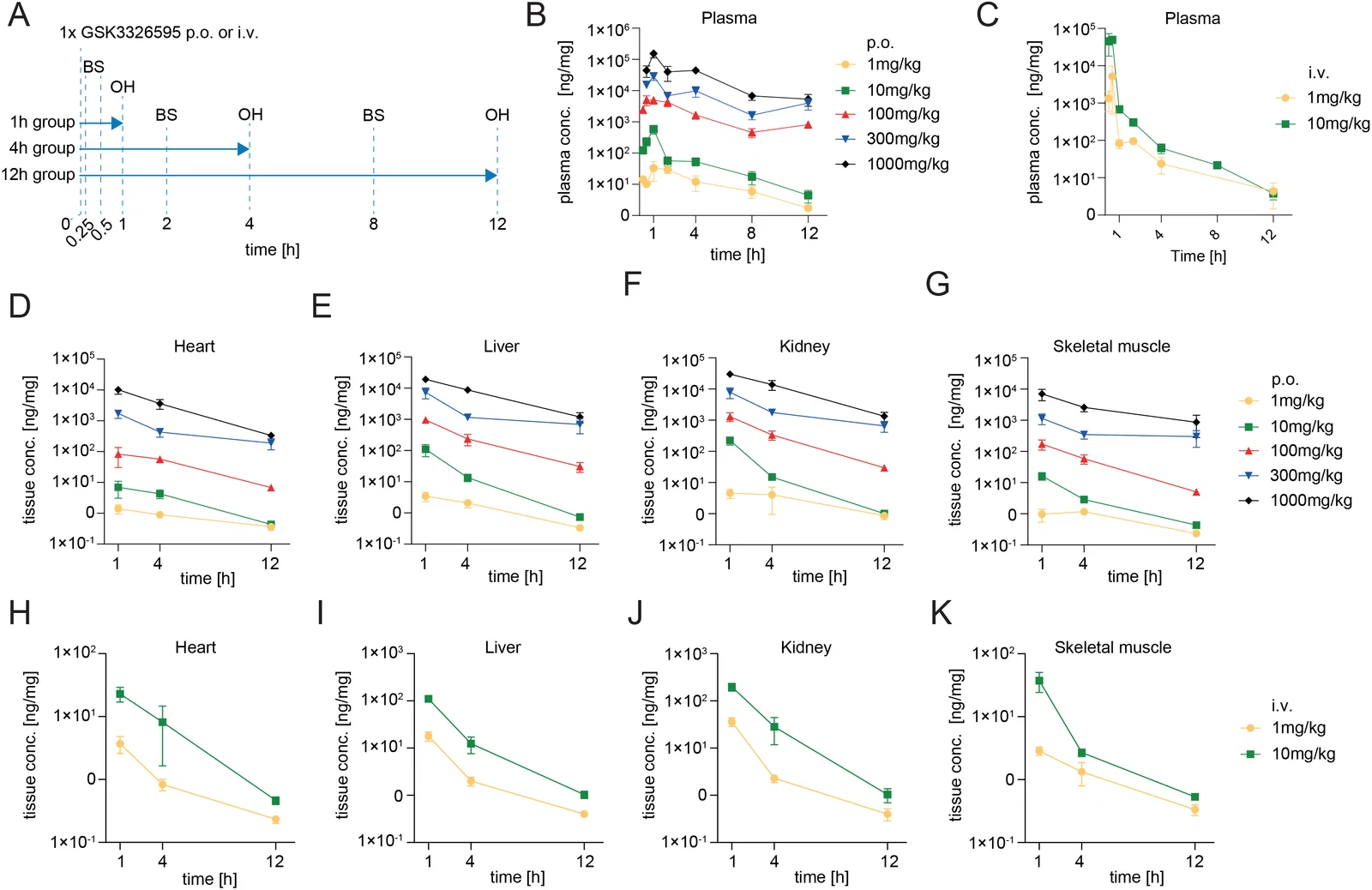
Pharmacokinetic profiling of GSK3326595 *in vivo*. (A) Schematic representation of the study. (B, C) Mass spectrometric analyses of GSK3326595 levels in plasma upon oral (B) and intravenous (C) administration during 12 h. (D-K) Mass spectrometric analyses of GSK3326595 levels in different organs upon oral (D-G) and intravenous (H-K) administration during 12 h. The experiment was performed with n = 3 animals per group as defined in (A). Final data and n numbers per time point are indicated in Suppl. Table 1. (A) Schematic representation of the study. (B, C) Mass spectrometric analyses of GSK3326595 levels in plasma upon oral (B) and intravenous (C) administration during 12 h. (D-K) Mass spectrometric analyses of GSK3326595 levels in different organs upon oral (D-G) and intravenous (H-K) administration during 12 h. The experiment was performed with n = 3 animals per group as defined in (A). Final data and n numbers per time point are indicated in Suppl. Table 1.

Given the diverse role of PRMT5 across organ systems as well as the clinical relevance of PRMT5i strategies, we further characterized GSK3326595 in terms of its pharmacokinetic profile in mice. Upon a single-dose administration *via* oral gavage or intravenous injection inhibitor concentration was determined in plasma and various tissues over a course of 12 h (Fig. 2A, Suppl. Table 1). We found the plasma peak concentration 1 h post application indicating a rapid absorption along with an exponential decline by >90% on average over a course of 12 h (Fig. 2B, C). Similar kinetics were observed across a broad spectrum of applied dosages. The oral bioavailability was calculated as 5.70% along with a plasma half-life of 150 min.

Consistent with plasma, we found maximum levels of GSK3326595 in the liver, kidney, heart and skeletal muscle after 1 h (Fig. 2D-K). The clearance was thereby comparable to plasma. Overall, kidney and liver presented with the highest absolute inhibitor concentrations (1316 and 960 ng/mg, respectively, based on oral administration of 100 mg/kg) followed by skeletal muscle and the heart (172 and 82.7 ng/mg, respectively) indicating a broad distribution in different tissues (Suppl. Fig. 1A-E). This result is moreover consistent across a scale of dosages ranging from 1 mg/kg to 10 mg/kg and 1000 mg/kg for intravenous and oral administration, respectively. Thus, we conclude that GSK3326595 is distributed globally, particularly in eliminating organs while muscle tissue and especially the heart is only targeted by 26.0% and 19.0% dosage on average, respectively, indicating an altered pharmacokinetic profile. Using SDMA as readout, single-dose application moreover did not acutely induce reduced PRMT5 activity in left-ventricular tissue (Suppl. Fig. 1F).

### 2.3 GSK3326595 treatment *in vivo*

To test the tolerance of a short-term treatment in mice we performed a study including administration over 5 consecutive days. Based on the pharmacokinetic profile oral application was performed once or twice a day in order to maintain maximal inhibitor levels. We did not detect changes in ejection fraction and tissue morphology indicating no immediate detrimental effects or fibrotic remodeling under these conditions (Suppl. Fig. 2A-C, Suppl. Table 2). On the molecular level, SDMA levels showed moderately increased levels pointing towards a maintained and potentially compensatorily increased overall type II PRMT enzymatic activity in the heart upon a 5-days PRMT5i exposure (Suppl. Fig. 2D). Specifically, the methylation status of histone residues H3R2, H3R8 and H4R3 that have been attributed to PRMT5 before [15], [16], [17] was not altered (Suppl. Fig. 2E).

**Fig. 3:**
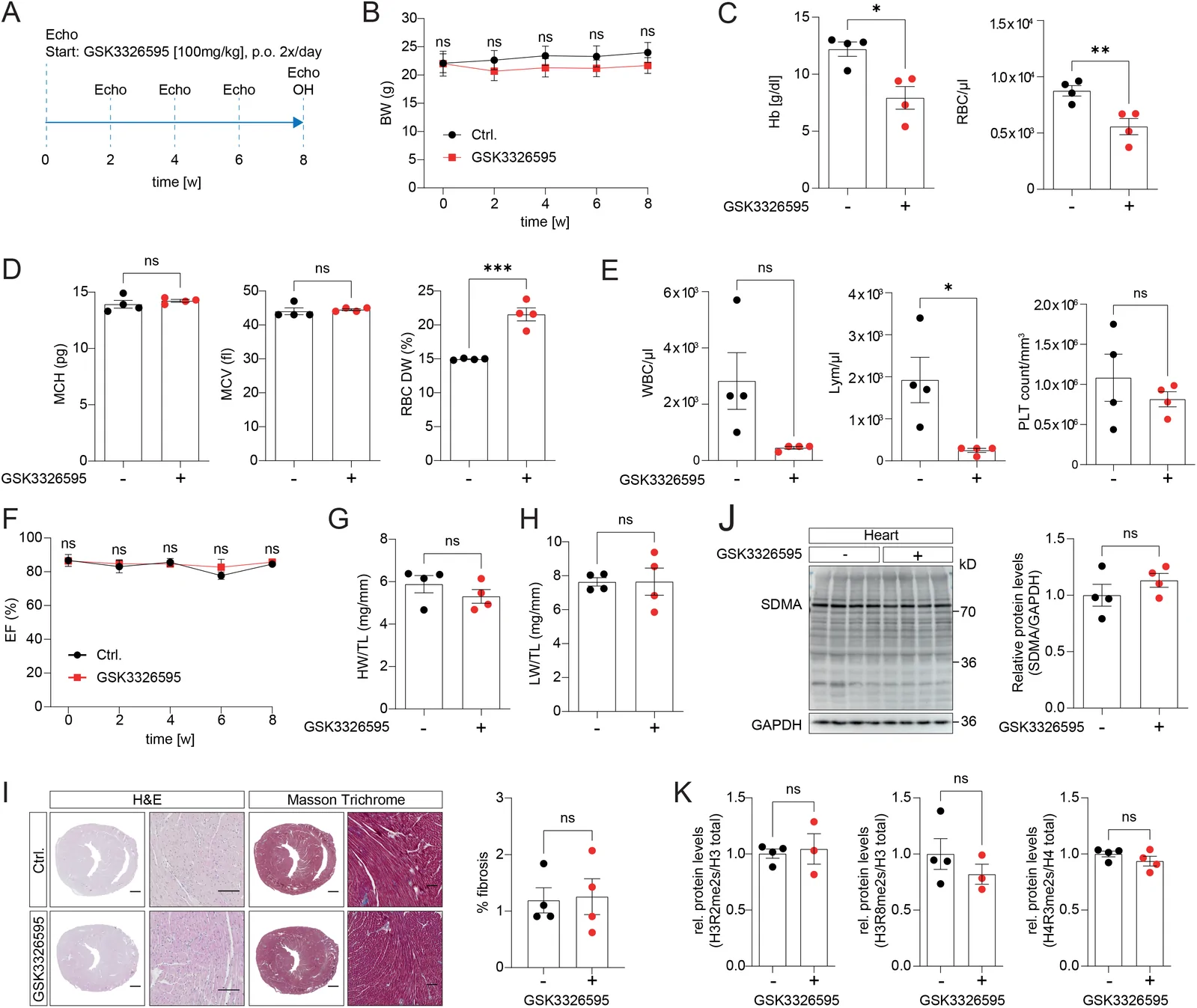
Long-term application of GSK3326595 in mice. (A) Schematic representation of the study conducted over the course of 8 weeks (w). The experiment was performed with *n* = 4 animals per group. (B) Longitudinal analysis of the body weight during long-term administration of GSK3326595. Statistical analysis: values are presented as Mean ± SEM (repeated-measures two-way ANOVA with the Geisser-Greenhouse correction and Bonferroni's multiple comparisons test; no statistical significance was found for any of the indicated time points). (C-E) Blood cell parameters of PRMT5i-treated and control animals (Hemoglobin, Hb; Red Blood Cells, RBC; Mean Corpuscular Hemoglobin, MCH; Mean Corpuscular Volume, MCV; Red Blood Cell Distribution Width, RBC DW; White Blood Cells, WBC; Lymphocytes, Lym; Platelets, PLT). Statistical analysis: values are presented as Mean ± SEM, *p = 0.0109 (Hb), **p = 0.0098 (RBC), ***p = 0.0005 (RBC DW), *p = 0.0215 (Lym) (unpaired *t-*test). (F) Echocardiographic analysis (ejection fraction, EF) of PRMT5i-treated mice over the course of 8 weeks. Statistical analysis: values are presented as Mean ± SEM (repeated-measures two-way ANOVA with the Geisser-Greenhouse correction and Bonferroni's multiple comparisons test; no statistical significance was found for any of the indicated time points). (G, H) Heart weight to tibia length ratio (HW/TL) and lung weight to tibia length ratio (LW/TL) after long-term administration of GSK3326595. Statistical analysis: values are presented as Mean ± SEM (unpaired t-test). (I) Histological analysis of cardiac cross-sections. Bars represent 500 μm and 100 μm, respectively. Statistical analysis: values are presented as Mean ± SEM (unpaired *t-*test). (J, K) Immunoblot analyses of global SDMA and histone targets of PRMT5 in left-ventricular tissue after GSK3326595 application for 8 weeks. Statistical analysis: values are presented as Mean ± SEM (unpaired *t-*test).

Given the clinical relevance of long-term PRMT5i regimes in patients [18] we extended our model to an 8-weeks experiment in order to recapitulate maintained antineoplastic therapy (Fig. 3A). Overall, long-term administration was well tolerated as determined by stable body weight without treatment-associated mortality (Fig. 3B). However, in agreement with reported clinical data, significant anemia and lymphopenia were found in PRMT5i-treated mice indicating that PRMT5 is required for sufficient hematopoiesis (Fig. 3C-E). In contrast, platelets counts were unaltered. Remarkably, despite major effects on blood cells, systolic cardiac function was preserved over the course of 8 weeks indicating that the heart mainly resists pharmacological PRMT5i although this study has limited power to exclude modest adverse effects (Fig. 3F). Consistently, further biometric parameters such as heart weight-to-tibia length and lung weight-to-tibia length did not reveal PRMT5i-induced alterations in terms of cardiac hypertrophy and lung congestion (Fig. 3G, H). On a morphological level, myocardial fibrosis remained stable as determined by Masson Trichrome staining (Fig. 3I). Along this line, global SDMA patterns did not change indicative of preserved PRMT5 activity (Fig. 3J). Further readouts included histone modifications such as H3R2me2s, H3R8me2s and H4R3me2s (Fig. 3K).

**Fig. 4:**
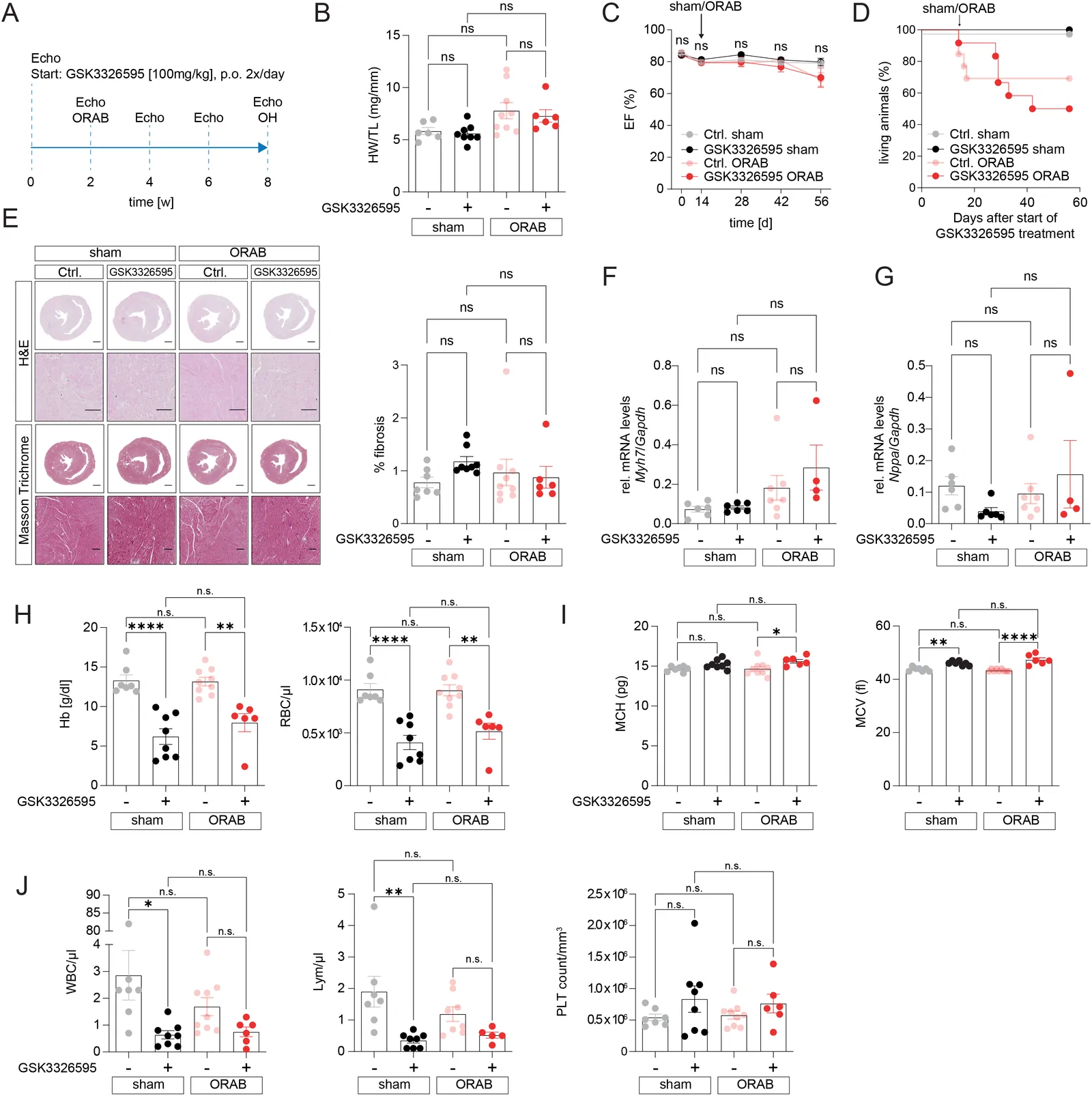
PRMT5i in a cardiac stress model. (A) Schematic representation of the study conducted over the course of 8 weeks (w). (B) Analysis of heart weight (HW) of GSK3326595-treated and control animals 6 weeks after ORAB or sham surgery. Values were normalized to tibia length (TL). Statistical analysis: values are presented as Mean ± SEM (Ordinary one-way ANOVA and Tukey‘s multiple comparisons test). (C) Longitudinal echocardiographic analysis of PRMT5i-treated mice during the course of 56 days (d) combined with ORAB/sham surgery. Statistical analysis: values are presented as Mean ± SEM (Mixed-effects model with the Geisser-Greenhouse correction and Bonferroni's multiple comparisons test; no statistical significance was found for any of the indicated time points in any group comparison). (D) Mortality analysis during long-term pharmacological treatment and surgery. (E) Histological analysis of cardiac cross-sections. Bars represent 500 μm and 100 μm, respectively. Statistical analysis: values are presented as Mean ± SEM (Ordinary one-way ANOVA and Tukey's multiple comparisons test). (F, G) Quantitative PCR on *Myh7* (F) and *Nppa* expression (G) in left-ventricular tissue. Values were normalized to *Gapdh*. Statistical analysis: values are presented as Mean ± SEM (Ordinary one-way ANOVA and Tukey's multiple comparisons test). (H-J) Blood cell parameters of PRMT5i-treated and control animals upon ORAB/sham surgery (Hemoglobin, Hb; Red Blood Cells, RBC; Mean Corpuscular Hemoglobin, MCH; Mean Corpuscular Volume, MCV; White Blood Cells, WBC; Lymphocytes, Lym; Platelets, PLT). Statistical analysis: values are presented as Mean ± SEM, ****p < 0.0001, **p = 0.0011 (Hb), ****p < 0.0001, **p = 0.0011 (RBC), *p = 0.0472 (MCH), **p = 0.0019 ****p < 0.0001 (MCV), *p = 0.0173 (WBC), **p = 0.0030 (Lym) (Ordinary one-way ANOVA and Tukey‘s multiple comparisons test). The experiment was performed with *n* = 7 (Ctrl. sham), *n* = 8 (GSK3326595 sham), *n* = 9 (Ctrl. ORAB) and *n* = 6 (GSK3326595 ORAB) animals per group.

Since cancer and cardiovascular disease are associated, particularly in patients of higher age [19] we set out to further mimic the simultaneous coincidence of anti-cancer therapy and cardiac stress by combining anti-PRMT5 therapy with a model of cardiac pressure overload (Fig. 4A). Specifically, we used mild aortic constriction *via* O-ring aortic banding (ORAB) [20] to induce subclinical burden along with subsequent adaptive hypertrophy but compensated systolic function. As expected, ORAB induced modest cardiac hypertrophy along with preserved contractile parameters (Fig. 4B, C). Overall mortality was equally reduced upon ORAB (Fig. 4D), however while control animals undergoing ORAB died during or shortly after surgery, most PRMT5i-treated mice showed delayed mortality starting 2 weeks post-surgery which might point to a dysfunctional long-term adaptability to cardiac stress, particularly given the overt effects on hematopoiesis. Histological analysis illustrated no significant fibrotic remodeling (Fig. 4E) along with stable expression of classical cardiac stress markers such as *Myh7* and *Nppa* (Fig. 4F,G). In line with the previous long-term study, chronic PRMT5i induced significant normocytic normochromic anemia and leucopenia while platelets were not affected indicating major effects on bone marrow function (Fig. 4H-J). Conclusively, this study indicates that the PRMT5 inhibitor GSK3326595 does not induce overt adverse cardiac effects in mice under the conditions tested. However, major effects on hematopoiesis are manifested and need to be critically considered, particularly when assessing global parameters such as mortality.

## 3 Discussion

Considering complex conditions such as neoplasms, cardiovascular diseases and neurological disorders not only as independent entities but rather as highly dynamic syndromes along with both, upstream and downstream impact from and on the entire organism is a major achievement of current research. Along this line, disentangling pathophysiologically causative molecular mechanisms from secondary (mal-)adaptive alterations represents a critical task of biomedical research and immediately translates into clinical practice often dealing with the simultaneous or successive management of several comorbidities. As such, cardio-oncology has gained particular relevance given the high prevalence of both, malign neoplasms as well as cardiovascular entities. Many chemotherapeutics entail cardiotoxic potential including anthracyclines, trastuzumab and immune checkpoint inhibitors [21]. Given the rapid progress in identifying novel therapeutic targets and strategies, unraveling adverse potential often remains challenging for both, clinical care and basic science.

Here, we focus on PRMT5 as a promising target of antineoplastic therapies, in particular hematological entities. Inhibiting PRMT5's methyltransferase activity was shown to effectively interfere with tumor growth *in vitro* and *in vivo* [7], [8], [22] while genetic models of PRMT5 deficiency point towards essential properties in cardiomyocytes raising the question if systemically targeting PRMT5 might induce cardiac impairment.

Unexpectedly, while a strong impact on blood cells, especially erythrocytes was recapitulated, pharmacological PRMT5i did not alter cardiac performance acutely and within chronic exposure for 8 weeks and, thus, did not mimic previously reported cardio-depressive consequences. Pharmacokinetics indicated uptake into left-ventricular tissue, although absolute values were significantly lower than in proliferating organs such as the liver. We therefore conclude that GSK3326595 predominantly targets tissues and cells with high proliferative activity which was indeed recapitulated *in vitro* when comparing HEK293 cells and primary cardiomyocytes. In addition to pharmacokinetic properties, mitotic capacity itself correlates with PRMT5 enzymatic activity [23]. Hence, it needs to be considered carefully if and to which extent increased enzyme activity might favor a more effective pharmacological targeting thereby complementing pharmacokinetic and pharmacodynamic aspects. Addressing the impact of PRMT5i on functional states of complex organs, these differential effects culminate in additive, synergistic or antagonistic effects based on cell type composition. Specifically for the heart, immediate detrimental effects on systolic function, tissue integrity and molecular parameters were not observed in this study. Along this line, manifested adverse cardiovascular events were not described in clinical studies of GSK3326595 [12], [18] although a particular cardiological examination and follow-up was not reported. Our results might thereby especially point to increased susceptibility of patients at high cardiovascular risk with present or previous cardiac impairment.

## 4 Limitations of the study

Although we performed extensive profiling, we acknowledge that pharmacokinetics and pharmacodynamics of GSK3326595 in humans and mice, particularly under persistent tumor conditions might differ. Hence, further studies are required to evaluate the impact of manifest malignant neoplasms on systemic pharmacological properties and to relate the obtained data to drug levels in patients. Since cardiotoxicity is particularly overt in combination therapies, additional studies might therefore evaluate the cardio-detrimental potential of GSK3326595 as additive to established anti-cancer regimes, the mutual impact of and on extra-cardiac effects such as suppression of hematopoiesis, and by additional cardiac parameters such as diastolic function, arrhythmia and subtle injury that are not addressed in this study. These aspects further translate to the molecular level. Despite established surrogate parameters such as SDMA and histone modifications, evidence of PRMT5 specificity often remains inconclusive, specifically against the background of potential compensatory effects of other type II PRMTs.

## CRediT authorship contribution statement

**Victoria Mauz:** Writing – review & editing, Writing – original draft, Visualization, Validation, Project administration, Investigation, Formal analysis, Data curation, Conceptualization. **Clara Steinacher:** Writing – review & editing, Investigation, Formal analysis. **Jürgen Burhenne:** Writing – review & editing, Resources, Methodology, Investigation, Formal analysis, Data curation. **Kevin Steimel:** Writing – review & editing, Methodology, Investigation, Formal analysis. **Christina Mertens:** Writing – review & editing, Resources, Methodology, Investigation, Formal analysis, Data curation. **Martina U. Muckenthaler:** Writing – review & editing, Supervision, Resources. **Matthias Dewenter:** Writing – review & editing, Validation, Investigation, Formal analysis, Data curation. **Johannes Backs:** Writing – review & editing, Writing – original draft, Supervision, Resources, Funding acquisition, Conceptualization.

## Funding

This work was supported by grants from the 10.13039/501100001659Deutsche Forschungsgemeinschaft (Collaborative Research Center CRC1550 “Molecular Circuits of Heart Disease,” INST 35/1699-1) to J.B., the 10.13039/100010447DZHK (German Centre for Cardiovascular Research) from the 10.13039/501100002347BMBF (German Ministry of Education and Research) to J.B., and from the MWK-BW (Ministry for Science and Culture of the federal state Baden-Württemberg: Innovation Campus Rhine-Neckar Region - Expansion of Cooperation and Translation, Promotion of the Cardiovascular Focus). The funders had no role in study design, data collection and analysis, the decision to publish or preparation of the manuscript.

## Declaration of competing interest

The authors declare no competing interests.

## Appendix A

### A.1 Methods

#### A.1.1 *In vivo* studies

All animal studies were approved by and conducted in accordance with the German Animal Welfare Act and approved by the Animal Experiment Review Board of the Regierungspräsidium Karlsruhe, Germany. The study refers to the application G210-19. C57BL/6N mice were purchased from Charles River at the age of 12 weeks. Male and female mice were included in an equal ratio.

#### A.1.2 Animal procedures

##### A.1.2.1 Application of GSK3326595

The compound (Medchemexpress) was dissolved in 5% DMSO, 40% PEG300, 5% Tween-80 in isotonic saline solution and administered either via oral gavage or by injection into the tail vein. Control animals received the same solution without compound.

##### A.1.2.2 Transthoracic echocardiography

Cardiac performance was determined by transthoracic echocardiography. Measurements on conscious mice were performed using a Vevo® 2100 system with a 55 MHz transducer (Visualsonics). Ejection fraction (EF) was averaged over at least 5 contraction cycles. The experimenter was blinded for genotype and treatment.

##### A.1.2.3 O-Ring Aortic Banding (ORAB)

The surgical procedure was essentially performed as previously described^20^. In brief, animals were narcotized using Isoflurane. The thorax was opened and a nitrile O-ring (inner diameter 0.020 inch) was closed around the aorta ascendens in order to enhance pressure load of the left ventricle. Perisurgically and for the following days, analgesia was maintained via buprenorphine.

#### A.1.3 Mass spectrometry and blood parameters

GSK3326595 concentrations in murine plasma and tissues were determined by UPLC-MS/MS using EPZ015666 as internal standard. Stock solutions and calibration/quality control solutions of GSK3326595 and EPZ015666 were prepared in 1:1 H_2_O/ACN. Tissue samples were homogenized in water using a Bead Ruptor 4 homogenizer (Omni International Inc, USA) for at least 120 s with ceramic beads. After protein precipitation with acetonitrile, plasma and tissue sample extracts were measured using an UPLC-MS/MS (Waters, USA) setup including an Acquity UPLC I-Class® coupled with a Xevo TQ-S triple stage quadrupole mass spectrometer (Waters), which featured a heated ESI source (Z-Spray in the positive MRM mode). Gradient chromatography was conducted using an ACQUITY UPLC BEH C18 column (130 Å, 1.7 μm, 2.1 mm × 50 mm; Waters, USA) consisting of eluent A (95/5 H2O/ACN with 0.1% formic acid) and eluent B (ACN with 0.1% formic acid). GSK3326595 calibration range was determined between 5 and 10,000 ng/mL and ng/g for plasma and tissue homogenate, respectively.

Hematological parameters were analyzed using an automated hematology analyzer (ScilVet ABC, ABX Diagnostics, France) according to manufacturer’s instructions. Whole blood samples were collected into EDTA-coated tubes to prevent coagulation. The following parameters were determined: red blood cell count (RBC), white blood cell count (WBC), hemoglobin concentration (HGB), mean corpuscular volume (MCV), mean corpuscular hemoglobin (MCH), platelet count (PLT), and differential leukocyte counts.

#### A.1.4 *In vitro* studies

HEK293 cells (ATCC) were cultured in DMEM (PanBiotech) supplemented with 10% FCS (Thermo Fisher) and 1% penicillin-streptomycin (Thermo Fisher) and incubated at standard conditions. Cells between passage 5 and 18 were used for experiments. Neonatal rat ventricular myocytes (NRVMs) were isolated from 24h-to-72h old rats using the Neonatal Heart Dissociation Kit (Miltenyi Biotec) according to manufacturer’s recommendations, cultured in DMEM supplemented with 10% FCS and 1% penicillin-streptomycin and incubated at standard conditions. EPZ015666 and GSK3326595 (MedChemExpress) were dissolved in DMSO and added into the culture medium at indicated dosages. For protein analysis, cells were treated for 96 h including a medium exchange after 48 h. Control cells were treated with DMSO accordingly. Single values in the analysis represent individually treated wells (technical replicates) and experiments were reproduced at least three times using independent cell passages or preparations.

PRMT5i-associated cytotoxicity was assessed using the CellTiter 96 Cell Proliferation Assay (Promega, G4000) according to manufacturer’s recommendations upon 48 h exposure to either EPZ015666, GSK3326595 or the respective DMSO control. The treatment was performed in individual wells and absorbance at 570 nm was recorded on an EnSight Plate reader (PerkinElmer).

#### A.1.5 Immunoblotting

Murine tissues and cultured cells were homogenized and lysed in Kranias buffer (30 mM Tris-HCl pH 8.8, 5 mM EDTA, 30 mM NaF, 3% SDS, 10% Glycerol) containing protease and phosphatase inhibitor cocktail (Sigma). Protein lysates were separated by SDS-PAGE and transferred onto nitrocellulose membranes (Amersham). Upon antibody probing, chemiluminescence was detected based on a Fusion FX system (Vilber). Band intensities were quantified with ImageJ (1.52a) [24].

Primary antibodies:

Anti-Dimethyl-Arginine antibody, symmetric SYM10 (Merck/Millipore, 07-412)
Anti-Histone H3R2me2s (abcam, ab194684)
Anti-Histone H3R8me2s (Epigentek, A-3706)
Anti-Histone H3 (Merck/Millipore, H9289)
Anti-Histone H4R3me2s (abcam, ab5823)
Anti-Histone H4 (Merck/Millipore, 07-108)
Anti-GAPDH antibody (Merck/Millipore, MAB374)

Secondary antibodies:

Goat Anti-Mouse (SouthernBiotech, 1031-05)
Goat Anti-Rabbit (SouthernBiotech, 4050-05)

#### A.1.6 RNA extraction and quantification

Left-ventricular tissue was lysed in TRIzol (ThermoFisher Scientific, 15596026) and total RNA was isolated using Chloroform/Isopropanol extraction according to manufacturer’s recommendations. 1µg of RNA was reverse transcribed (PrimaDIRECT 2x PCR Master Mix Kit (Steinbrenner)) and cDNA was subjected to quantitative PCR on a LightCycler480 (Roche) using SYBR Green Master Mix (Thermo Fisher).

Primer sequences:

mmMyh7:

For: 5’ AGC ATT CTC CTG CTG TTT CCT 3’
Rev: 5’ CCA GGC CTG TAG AAG AGC TG 3’

mmNppa:

For: 5’ ATT GAC AGG ATT GGA GCC CAG AGT 3’
Rev: 5’ TGA CAC ACC ACA AGG GCT TAG GAT 3’

mmGapdh:

For: 5’ GGT GGA CCT CAT GGC CTA CA 3’
Rev: 5’ CTC TCT TGC TCA GTG TCC TTG CT 3’

#### A.1.7 Histology

Heart cross-sections (2-chamber view) were fixed in Roti Histofix (Roth, P087.5) and dehydrated in ethanol. After paraffin embedding, slices of 6µm thickness were cut and used for Hematoxylin/Eosin and Masson’s trichrome staining. Imaging was performed on a Zeiss Axioscan 7. For analysis, 10 snapshots of fixed dimension were taken per animal and the percentage of fibrotic tissue was assessed based on color threshold using ImageJ.

#### A.1.8 Statistical analysis

Data sets are displayed as mean ± SEM. GraphPad Prism 9 software was used for statistics and visualization. Data sets were evaluated by either One-Way ANOVA and Tukey’s multiple comparisons test with multiplicity adjusted P values or unpaired t-test. If not otherwise indicated, p < 0.05 was considered as significant.

## Appendix B Supplementary data

Supplementary Fig. 1 Extended pharmacokinetic data. (A-E) Mass spectrometric analyses of GSK3326595 levels across different tissues upon single dose oral administration of indicated dosages. The experiment was performed with *n* = 3 animals per group. Tissues were analyzed 1 h after application. (F) Immunoblot analyses of left-ventricular tissue upon GSK3326595 application at indicated dosages. Statistical analysis: values are presented as Mean ± SEM ***p* = 0.0045, ****p* = 0.0004, **p* = 0.0185 (Ordinary one-way ANOVA and Dunnett‘s multiple comparisons test).

Supplementary Fig. 2 Short-term application of GSK3326595 in mice. (A) Schematic representation of the study. (B) Echocardiographic analysis of PRMT5i-treated mice before and after short-term application. Applications were performed either once or twice per day. The experiment was performed with n = 3 animals per group along with one animal (10 mg/kg) that died during the course of the experiment. Statistical analysis: values are presented as Mean ± SEM (repeated-measures two-way ANOVA with Bonferroni's multiple comparisons test). (C) Histological analysis of cardiac cross-sections. Bars represent 500 μm and 100 μm, respectively. Statistical analysis: values are presented as Mean ± SEM (Ordinary one-way ANOVA and Dunnett‘s multiple comparisons test). (D) Immunoblot analyses of global SDMA levels in left-ventricular tissue upon GSK3326595 application twice per day for 5 consecutive days at indicated dosages. Statistical analysis: values are presented as Mean ± SEM **p* = 0.0363, ***p* = 0.0029 (10 mg/kg), ***p* = 0.0012 (30 mg/kg), ***p* = 0.0023 (100 mg/kg) (Ordinary one-way ANOVA and Dunnett‘s multiple comparisons test). (E) Immunoblot analyses of histone targets of PRMT5 in left-ventricular tissue. Statistical analysis: values are presented as Mean ± SEM **p* = 0.0472 (Ordinary one-way ANOVA and Dunnett‘s multiple comparisons test).

Supplementary Table 1 Pharmacokinetic data sets.

Supplementary Table 2 Echocardiographic data sets.
