# Supplementary material for: Systemic administration of the PRMT5 inhibitor GSK3326595 does not induce overt cardiotoxicity in the murine heart
Source: J Mol Cell Cardiol Plus. 2026 Aug 15;18:100863. doi: 10.1016/j.jmccpl.2026.100863 (PMC13594947; doi:10.1016/j.jmccpl.2026.100863)

Suppl. Figure 1

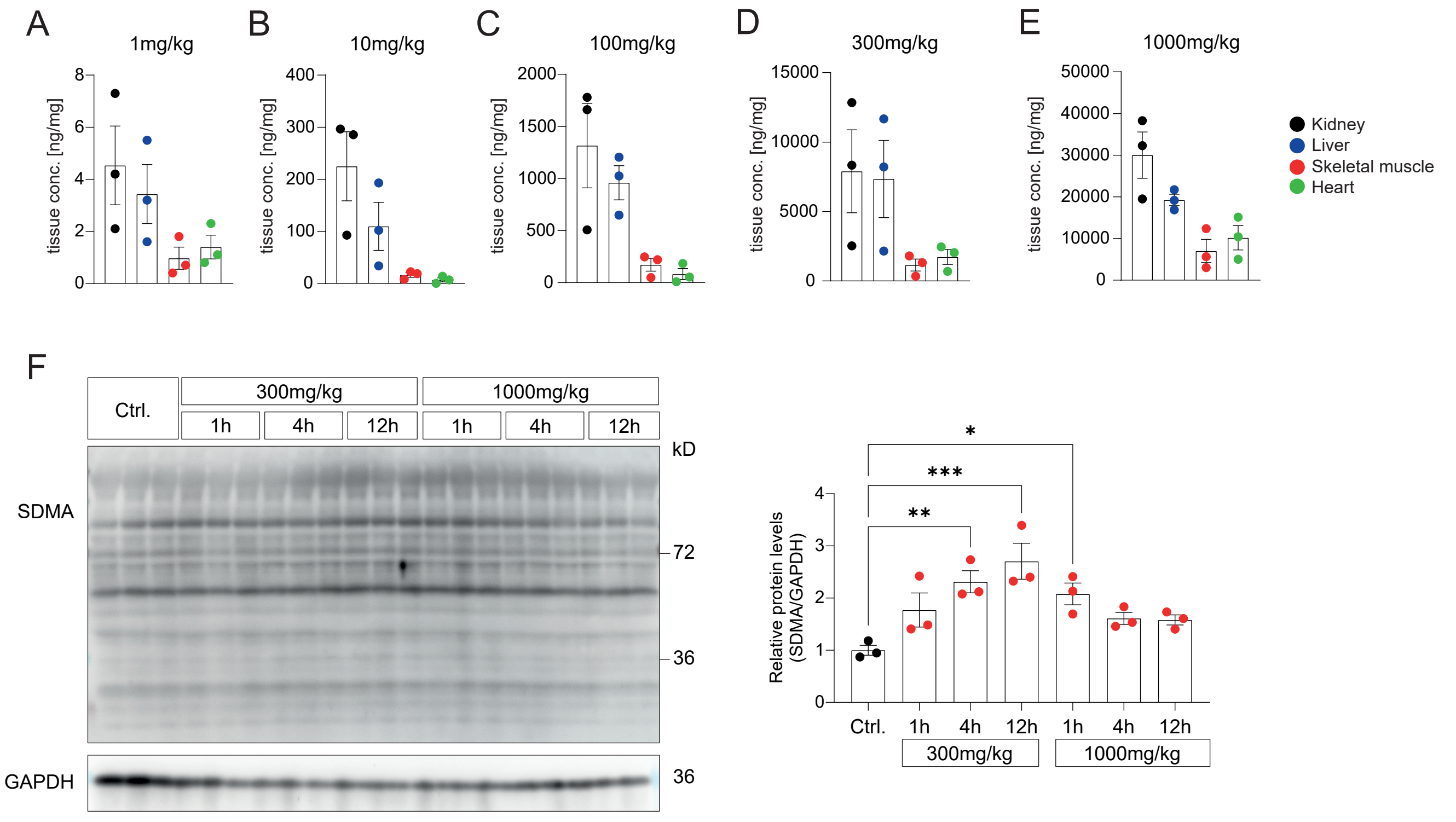

Supplement: Supplementary file 1 [file mmc1.pdf]
